# *Rhomboid* Enhancer Activity Defines a Subset of *Drosophila* Neural Precursors Required for Proper Feeding, Growth and Viability

**DOI:** 10.1371/journal.pone.0134915

**Published:** 2015-08-07

**Authors:** Amy L. Gresser, Lisa M. Gutzwiller, Mackenzie K. Gauck, Volker Hartenstein, Tiffany A. Cook, Brian Gebelein

**Affiliations:** 1 Division of Developmental Biology, Cincinnati Children’s Hospital Medical Center, Cincinnati, Ohio, United States of America; 2 Department of Molecular, Cell and Developmental Biology, University of California Los Angeles, Los Angeles, California, United States of America; 3 Department of Pediatric Ophthalmology, Cincinnati Children’s Hospital Medical Center, Cincinnati, Ohio, United States of America; Indiana University, UNITED STATES

## Abstract

Organismal growth regulation requires the interaction of multiple metabolic, hormonal and neuronal pathways. While the molecular basis for many of these are well characterized, less is known about the developmental origins of growth regulatory structures and the mechanisms governing control of feeding and satiety. For these reasons, new tools and approaches are needed to link the specification and maturation of discrete cell populations with their subsequent regulatory roles. In this study, we characterize a *rhomboid* enhancer element that selectively labels four *Drosophila* embryonic neural precursors. These precursors give rise to the hypopharyngeal sensory organ of the peripheral nervous system and a subset of neurons in the deutocerebral region of the embryonic central nervous system. Post embryogenesis, the *rhomboid* enhancer is active in a subset of cells within the larval pharyngeal epithelium. Enhancer-targeted toxin expression alters the morphology of the sense organ and results in impaired larval growth, developmental delay, defective anterior spiracle eversion and lethality. Limiting the duration of toxin expression reveals differences in the critical periods for these effects. Embryonic expression causes developmental defects and partially penetrant pre-pupal lethality. Survivors of embryonic expression, however, ultimately become viable adults. In contrast, post-embryonic toxin expression results in fully penetrant lethality. To better define the larval growth defect, we used a variety of assays to demonstrate that toxin-targeted larvae are capable of locating, ingesting and clearing food and they exhibit normal food search behaviors. Strikingly, however, following food exposure these larvae show a rapid decrease in consumption suggesting a satiety-like phenomenon that correlates with the period of impaired larval growth. Together, these data suggest a critical role for these enhancer-defined lineages in regulating feeding, growth and viability.

## Introduction

Properly controlled organismal growth involves a delicate balance between nutrient consumption and utilization. Achieving this balance requires the precise yet adaptable regulation of many interrelated physiological processes that can be divided into three broad categories: food intake, metabolism and nutrient usage. While food intake may be the easiest to conceptually understand, the factors underlying an organism’s decision to begin or cease feeding are varied and complex. These include environmental cues such as food availability, sensory stimuli and social norms, as well as intrinsic states of hunger or satiety, mediated in part by neuroendocrine feedback from metabolic and homeostatic pathways [1–4]. Understanding the mechanisms that regulate feeding and growth has long been an important focus of research but has recently gained greater urgency due to the dramatic surge in human obesity and related diseases.

*Drosophila melanogaster* offers numerous advantages as a model system to study growth regulation and has recently become an organism of choice for investigations of satiety [5–10]. An abundance of powerful genetic tools exist to manipulate gene expression in fruit flies and their short life cycle, in which all growth is confined to the larval period, makes alterations in growth easy to quantify. Furthermore, many of the signaling pathways that control size in the fly are highly analogous to, but often less redundant than, those found in vertebrates [4,11,12]. These include an array of neural and endocrine mechanisms that inextricably link growth control to nervous system development.

The *Drosophila* nervous system is derived from two populations of progenitor cells: neuroblasts, which give rise to the central nervous system (CNS), and sensory organ precursor cells (SOPs), which give rise to the peripheral nervous system (PNS). Each has been extensively characterized at a genetic level, but in many cases the relationship between the identity of individual precursor cells and their ultimate function is not clear.

In this study, we characterize a subset of neural precursor cells and correlate their development with control of feeding and growth. We previously identified a *cis*-regulatory element (enhancer) of the *rhomboid* (*rho*) serine protease, a catalyst for epidermal growth factor (*spitz*) secretion [13–15]. This enhancer (Rho654) contains four conserved regions (RhoA-D) that mediate activity in different parts of the embryo [13,16]. RhoA activates gene expression in a subset of abdominal SOPs leading to the induction of a hepatocyte-like oenocyte fate in neighboring cells. Here, we demonstrate that RhoB acts in four neural precursors within the embryonic head. Lineage tracing indicates these precursors give rise to two distinct structures in the late embryo, the hypopharyngeal sensory organ and a small subset of neurons in the deutocerebrum, a structure analogous to the vertebrate midbrain [17]. To ascertain the function of these neural precursors, we performed targeted toxin expression studies and observed defects in larval growth, developmental delay and/or lethality. Behavioral analyses revealed that the growth defect is correlated with transient decreases in feeding, suggesting a role for these cells in sensory feedback and regulation of satiation. Previous studies examining satiety in *Drosophila* [5–10] have focused primarily on adults. Our results suggest that satiation can also occur in larvae, and we introduce a paradigm for documenting such effects and define a new neural component in the regulation of feeding, growth and viability.

## Materials and Methods

### Fly stocks and immunostaining

*RhoAAA-Gal4* and *RhoBB-Gal4* constructs were created by cloning three copies of the RhoA element or two copies of the RhoB element (sequences as in [16]) into the *hs43-Gal4* P-element vector and confirmed by DNA sequencing. Transgenic lines were established in a *yw* ^*67*^ background using P-element transformation (Rainbow Transgenic Flies, Inc., Camarillo, CA, USA). Other fly lines: *Rho654-Gal4* [13], *ato-LacZ* ([38], gift of Yuh Nung Jan, UCSF, San Francisco, CA, USA), *rho* ^*7M43*^ ([41], gift of Gary Struhl, Columbia University, New York, NY, USA), *sens-LacZ* (5.9, [81], gift of Hugo Bellen, Baylor College of Medicine, Houston, TX, USA), *spi* ^*1*^ ([42],gift of Gary Struhl, Columbia University, New York, NY, USA), *UAS-DTI* ([49], gift of Hyung Don Ryoo, NYU, New York, NY, USA), *UAS-H2B-YFP* ([82], gift of Claude Desplan, NYU, New York, NY, USA), *UAS-CD8-GFP* (Bloomington Drosophila Stock Center (BDSC), Indiana University, Bloomington, IN, USA), *tub-Gal80* ^*ts*^ ([55], BDSC) and *yw* ^*67*^ (BDSC). All experiments were conducted at 25°C unless otherwise noted. Embryos were harvested, fixed and immunostained using standard protocols. Larvae were dissected in PBS and fixed overnight in methanol at -20°C prior to staining. Primary antibodies: Ato (rabbit, 1:500, 84F, [83], gift of Yuh Nung Jan), β galactosidase (chicken, 1:1000, ab9361, Abcam, Cambridge, MA, USA), Brp (mouse, 1:50, nc82, Developmental Studies Hybridoma Bank (DSHB)),Dpn (rabbit, 1:500, 44C,[84], gift of Jill Wildonger, University of Wisconsin-Madison, Madison, WI, USA), Elav (rat, 1:200, 7E8A10, DSHB), University of Iowa, Iowa City, IA, USA), Eys (mouse, 1:25, 21A6, DSHB), Fas2 (mouse, 1:75, 1D4, DSHB), Futsch (mouse, 1:50, 22C10, DSHB), GFP/YFP (rabbit, 1:500, A11122, Invitrogen, Carlsbad, CA, USA or goat, 1:500, AB6662, Abcam), HNF4 (guinea pig or rat, 1:1000, [18]), Ind (rabbit, 1:2000,[85], gift of Tonia Von Ohlen, Kansas State University, Manhattan, KS, USA), Msh (rabbit, 1:500, [86], gift of Chris Doe, University of Oregon, Eugene, OR, USA), Otd (guinea pig, 1:750, [87]), DPax2 (rat, 1:500, this paper), pERK (mouse, 1:50, anti-MAPK, Sigma-Aldrich, Inc., St. Louis, MO, USA), Pros (guinea pig, 1:500, [87]) and Vnd (rat, 1:1000, [88], gift of Ze’ev, Paroush, The Hebrew University, Jerusalem, Israel). Images were collected using an ApoTome-configured fluorescent microscope (Carl Zeiss Microscopy, LLC, Thornwood, NY, USA) or an A1R inverted confocal microscope (Nikon Instruments Inc., Melville, NY, USA).

### Antibody production

A DPax2 bacterial expression plasmid was created by cloning a PCR-amplified full length cDNA in-frame with an N-terminal 6-His tag (pET14b) and transformed into BL21-CodonPlus (DE3)-RP bacteria (Stratagene, Agilent Technologies, Santa Clara, CA, USA). Protein expression was induced using 0.25 mM IPTG for 2 hours. Cells were lysed in 8 M urea lysis buffer (ULB: 100 mM NaH2PO4, 10 mM Tris pH8.0, 10 mM imidazole, 8 M urea, 0.5% Igepal) and centrifuged for 30 minutes at 16,000 g. The supernatant was mixed with Ni-NTA beads (Qiagen, Inc., Valencia, CA, USA) for 2 hours at room temperature. Beads were washed three times with ULB and the protein was eluted in ULB plus 250 mM imidazole. The protein was tested for purity using SDS-PAGE and Coomassie blue gel staining and used to generate antibodies in a rat (Cocalico Biologicals, Inc., Reamstown, PA, USA).

### Growth and spiracle eversion

Embryos were collected over a two hour period and maintained on apple agar plates supplemented with yeast paste. At 48, 78 and 89 hours after egg laying (AEL) or after pupariation, larvae/pupae were removed from plates, washed with distilled water to remove residual yeast paste and transferred to chilled microscope slides. Length was scored for 50 larvae/pupae. Spiracle eversion was scored for 5 replicates of 10 pupae. Data were compared using one way ANOVA with planned comparison of means.

### Developmental timing and viability

For non-temperature-sensitive experiments, embryos were collected for two hours at 25°C in vials containing standard cornmeal agar food. Vials were scored twice daily for pupariation and eclosion. Data were compared using one way ANOVA with planned comparison of means. For Gal80^ts^-mediated temporally controlled experiments, embryos were collected for two hours in vials at 18°C or 29°C and either maintained at constant temperature throughout development or shifted to the opposite temperature at the conclusion of embryogenesis (~18 hours AEL at 29°C and ~41 hours AEL at 18°C). Vials were scored twice daily for pupariation and eclosion. Data were compared using the sign test (pupal formation) or one way ANOVA with planned comparison of means (survival, length of pupation, age at pupariation and eclosion).

### Wing size

Wings were dissected from one day old *Rho654>DTI;tub-Gal80* ^*ts*^ adults raised either at 18°C throughout development (no toxin expression) or at 29°C during embryogenesis and 18°C for the remainder of development (toxin expression during embryogenesis). Wings were dry mounted and imaged for auto-fluorescence using a Nikon A1R inverted confocal microscope. Wing areas were calculated using ImageJ [89]. Four wings were assayed for each gender, genotype and condition. Data were compared using one way ANOVA with planned comparison of means.

### Mouth hook morphology

Embryos were collected over a two hour period at 25°C and maintained on apple agar plates supplemented with yeast paste. Aged larvae were removed from the plates and washed with distilled water. Mouthhooks were dissected in PBS, transferred directly to mounting media, and imaged using a bright-field Zeiss microscope.

### Capacity of larvae to ingest and clear food

Embryos were collected over a two hour period on apple agar plates supplemented with yeast paste. At 72 hours AEL, larvae were removed from plates, washed with distilled water to remove residual yeast paste, starved for two hours on PBS-soaked filter paper and transferred to pre-warmed 6 cm diameter 2% agar plates supplemented with yeast paste containing blue food dye (McCormick & Company, Inc., Sparks, MD, USA). After one hour, larvae were removed and cleaned as before. Food intake was verified visually by the presence of blue coloring throughout the gut. Dye-consuming larvae were transferred to agar plates with non-dyed yeast paste, allowed to feed for an additional two hours and assayed for food clearance by the disappearance of blue coloring from the gut. Images were collected with a MicroPublisher 5.0 CCD camera (QImaging, Surrey, BC, Canada) connected to a MZ7.5 stereomicroscope (Leica Microsystems GmbH, Wetzlar, Germany) and iMac 5.1 (Apple, Cupertino, CA, USA) running QCapture version 3.1.2 software (QImaging).

### Triglyceride and glucose quantification

Embryos were collected over a two hour period on apple agar plates supplemented with yeast paste. At 72 hours AEL, larvae were removed from plates, washed with water to remove residual yeast paste, flash frozen and stored at -80°C. Larval samples were homogenized in 200 μl PBS with 0.05% Tween on ice and incubated at 70°C for 5 minutes. Measurement of triglyceride levels was performed using the Serum Triglyceride Determination Kit (TR0100, Sigma-Aldrich, Inc.). Thirty μl of larval samples or glycerol standards were incubated for 15 minutes at room temperature with 100 μl free glycerol reagent and assayed using a μQuant Microplate Spectrophotometer (BioTek Instruments, Inc., Winooski, VT, USA) at 540 nm. Samples were then incubated for an additional 15 minutes at room temperature with 25 μl triglyceride reagent and re-assayed at 540 nm. True triglyceride (“triglyceride”) concentrations were obtained by subtracting serum glycerol from total serum triglycerides. Determination of glucose levels was performed using the Glucose (HK) Assay Kit (GAHK-20, Sigma-Aldrich, Inc.). Ten μl of larval samples or glucose standards were incubated for 30 minutes at 37°C then for an additional 15 minutes at room temperature with 100 μl glucose reagent and assayed for absorbance at 340 nm. For each assay and genotype, three independent samples were assayed in triplicate and normalized to protein concentration. Total protein concentration for each sample was assayed in triplicate using Bio-Rad Protein Assay reagent (Bio-Rad Laboratories, Hercules, CA, USA) and compared to a BSA standard curve. Data were compared using one way ANOVA with planned comparison of means.

### Photophobicity

Larval photophobicity was tested as described previously [63] with minor modifications. Embryos were collected over a two hour period and maintained on apple agar plates supplemented with yeast paste. At 72 hours AEL, larvae were removed from plates, washed with distilled water to remove residual yeast paste and transferred to the boundary between the light and dark sides of a 10 cm 2% agar plate. Half of each plate (top and bottom) was covered with black electrical tape to create the dark side. Plates were lit from above using an Ace light source with EKE halogen lamp (Schott North America, Inc., Southbridge, MA, USA) set to 45% intensity. Larvae were allowed to wander for 15 minutes at room temperature after which the numbers on each half were counted. Four replicates of 20 larvae were scored and compared using the Cochran–Mantel–Haenszel test for repeated tests of independence.

### Food proximity and dye ingestion

Larvae were aged and washed as above. After one hour of starvation on PBS-soaked filter paper, larvae were transferred to pre-warmed 9 cm diameter 2% agar plates with a centered 3 cm spot of yeast paste containing blue food dye. Larvae were assayed in one of two conditions: transfer to periphery of feeding plate away from food source and transfer directly to the boundary of the food source. In both conditions, the number of larvae on or touching the yeast paste was scored at 5, 10, 30 and 60 minutes after placement and the number of larvae exhibiting any visible dye ingestion was scored after 60 minutes. Three replicates of 20 larvae were scored and compared using the Cochran–Mantel–Haenszel test for repeated tests of independence.

### Mouthhook and bodywall contractions

Larvae were aged, washed and starved for one hour as above. For food search assays, larvae were transferred individually to pre-warmed 9 cm diameter 2% agar plates and placed at a point 5 cm from a 1.5 cm radius semicircular spot of yeast paste and 2.5 cm from the plate edge. For food consumption assays, larvae were transferred individually to pre-warmed 9 cm diameter 2% agar plates spread with a thin coating of yeast paste. Mouthhook and bodywall contractions were scored for the same 25 larvae over 30 second intervals beginning at 0, 60, 150 and 270 seconds after transfer. Data were compared using one way ANOVA with planned comparison of means.

## Results

### *rho* enhancer activity defines precursors of the hypopharyngeal sensory organ and a deutocerebral neuron subset

**Fig 1:**
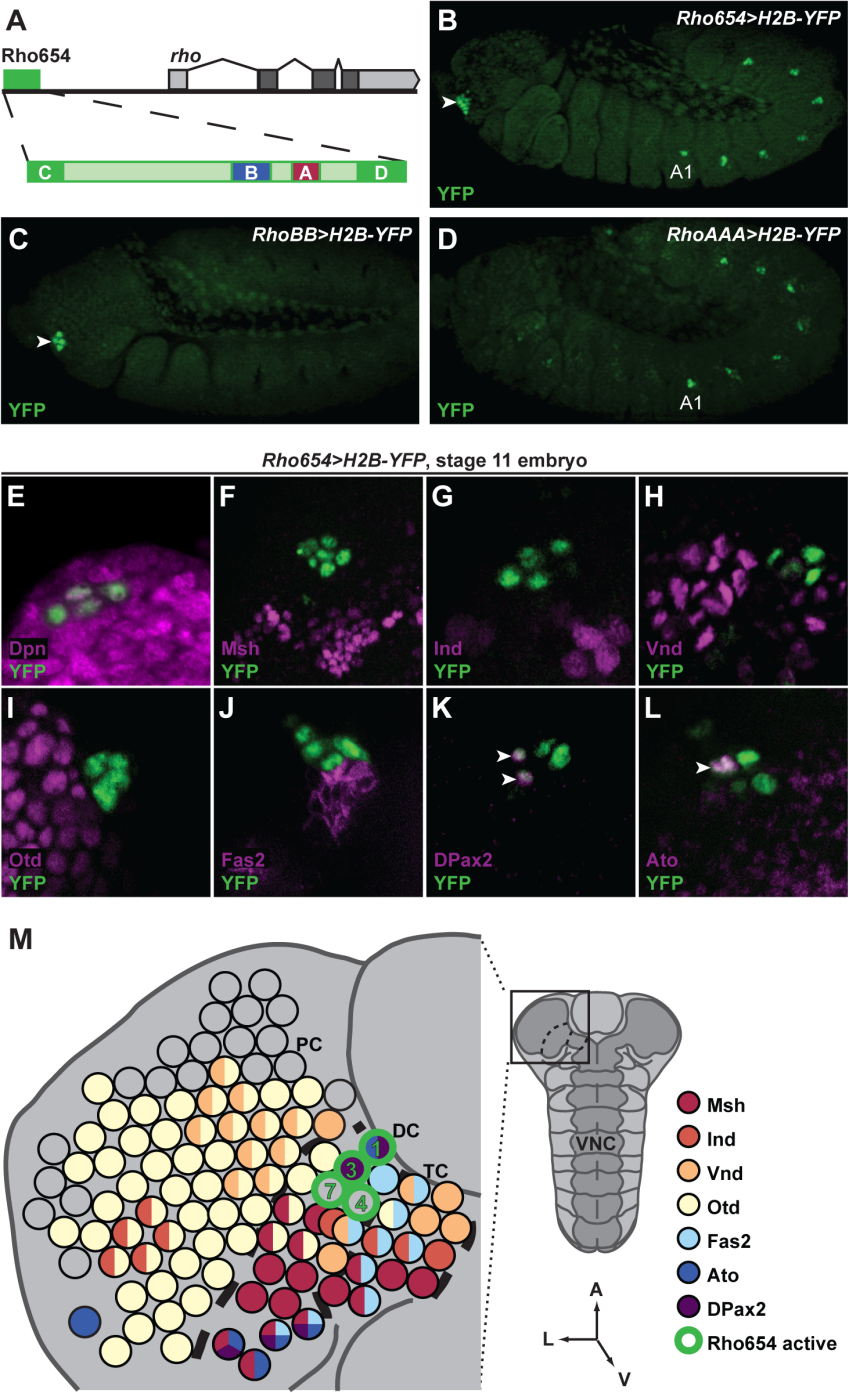
*rho* enhancer activity maps to four neural precursors in the embryonic head. (A) The *rho* locus highlighting the Rho654 enhancer’s four conserved elements (RhoA-RhoD). (B-D) Immunostaining of *Rho654>H2B-YFP* (B), *RhoBB>H2B-YFP* (C) and *RhoAAA>H2B-YFP* (D) reporter lines demonstrating that the RhoB region of Rho654 mediates activity in four neural precursors within the head (arrowheads in B and C) while the RhoA region acts in abdominal SOPs (A1 denotes first the first abdominal segment in B and D). Early (C) and slightly later (B,D) stage 11, *z*-projected lateral views. (E-M) Comparison of *Rho654>H2B-YFP* (E-L) with published expression data for CNS neuroblasts and SOPs (M, adapted from [17,90]). Co-expression of YFP with Dpn (E) indicates neural precursor identity. Lack of YFP co-expression with Msh (F), Ind (G), Vnd (H), Otd (I), or Fas2 (J) restricts Rho654 cell (green borders) identities to the Dv1/3 SOPs and Dv4/7 neuroblasts. Co-expression of YFP with DPax2 (K) and Ato (L) (arrowheads) is consistent with expression data for the Dv1/3 SOPs (M). Stage 11, *z*-projected ventral views with anterior up. PC: protocerebrum, DC: deutocerebrum, TC: tritocerebrum, VNC: ventral nerve cord. Bold dashed lines represent approximate neuromere boundaries.

The Rho654 enhancer drives *rho* expression in a subset of abdominal SOPs to induce the formation of oenocytes (Fig 1A and 1B) [13,16,18]. In addition, Rho654 acts in a bilaterally symmetric head cell cluster beginning at embryonic stage 11 (Fig 1B). Head activity is mediated by a conserved 68 bp region (RhoB; Fig 1A and 1C) that is distinct from the enhancer region (RhoA) responsible for abdominal activity (Fig 1A and 1D) [16]. A reporter consisting of two copies of the RhoB element (*RhoBB>H2B-YFP*) is sufficient to recapitulate Rho654 activity in the head (Fig 1C). Thus, Rho654 acts in two distinct cell populations in the *Drosophila* embryo: the RhoA element activates gene expression in abdominal SOPs while the RhoB element drives expression in the head.

At early stage 11, Rho654 activity labels a cluster of four cells in the head (Fig 1E). These cells co-stain with Deadpan (Dpn), a marker of both CNS neuroblasts and PNS SOPs [19], that labels approximately 100 neural precursors in each half of the developing head [20–22]. To determine in which neural precursors Rho654 acts, we examined a variety of markers that identify individual neuroblasts and SOPs [17,20,23–25]. Analysis of markers differentially expressed along the dorsal-ventral (*muscle segment homeobox* (*msh*, *Drop* [*Dr*]–Flybase) [26], *intermediate neuroblasts defective* (*ind*) [27] and *ventral nervous system defective* (*vnd*) [28]) (Fig 1F–1H) and anterior-posterior (*orthodenticle* (*otd*, *ocelliless* [*oc*]–Flybase) [29]) (Fig 1I) axes revealed that Rho654-defined cells lie near, but do not overlap with, cells expressing any of these factors. In addition, Rho654-labeled cells lie ventral to Fasciclin2 (Fas2)-positive cells [30] (Fig 1J). Using the nomenclature and neuroblast/SOP map proposed by Urbach et al. [25], we conclude that Rho654 acts in ventral deutocerebral neural precursors 1, 3, 4 and 7 (henceforth Dv1/3/4/7). Consistent with this conclusion, two Rho654-labeled cells express the *Drosophila* homolog of the vertebrate Pax2 factor (DPax2, *shaven* [*sv*]–Flybase) (Fig 1K), a marker associated with the Dv1/3 SOPs [17,31,32] and one expresses the proneural gene Atonal (Ato) [33] (Fig 1L) in agreement with prior characterization of Dv1 [25].

**Fig 2:**
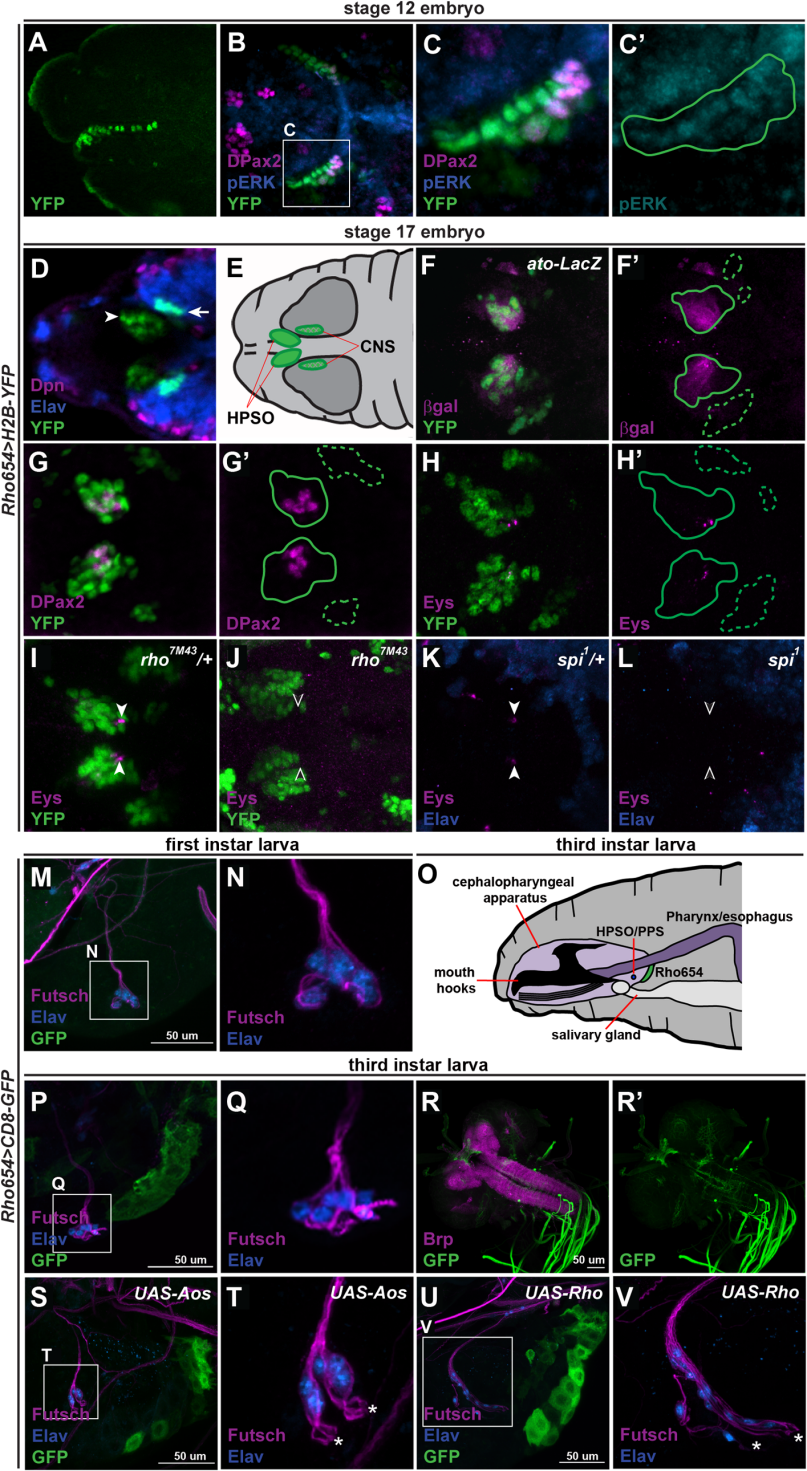
Fate mapping and characterization of labeled neural precursor progeny. (A-C) Lateral (A) and ventral (B-C) views of *Rho654>H2B-YFP*-labeled Dv1/3/4/7 progeny migrating posteromedially along the ventral pharynx at stage 12. Subsets of these cells express DPax2 and all express pERK (C, green outline denotes position of *Rho654>H2B-YFP*-labeled cells). *z*-projections with anterior to the left. (D-H) At stage 17, YFP-labeled cells form the HPSO (D-E, arrowhead) and a bilaterally symmetric subset of deutocerebral CNS neurons (D-E, arrow). Subsets of HPSO cells (solid outlines) express an *ato* reporter (F), DPax2 (G) and Eys (H). None of these markers is expressed in the YFP-labeled CNS-neurons (dashed outlines). *z*-projected dorsal views. (I-L) Expression of Eys remains in heterozygous *rho* ^*7M43*^ or *Spi* ^*1*^ mutants (I,K, arrowheads) but is lost in homozygotes (J,L, open arrowheads). Stage 17, *z*-projected dorsal views. (M-P) Rho654 reporter activity is not detectable in the pharyngeal epithelial region of first instar larvae (M-N) but is detectable by third instar (O-Q). The PPS (successor of the HPSO) is labeled by the neuronal markers Futsch (marked by monoclonal antibody 22C10) and Elav. *z*-projections lateral views with anterior to the left and dorsal up. (R) Rho654 reporter activity is also detected in a subset of midline cells in the supraesophageal CNS as well as a network of projections within the ventral nerve cord of third instar larvae. Bruchpilot (Brp, marked by nc82 antibody) defines the boundaries of the CNS neuropile. *z*-projected dorsal view. (S-T) Targeted overexpression of Aos alters PPS morphology, increasing the distance between Futsch-positive termini (asterisks, T) and Elav-positive nuclei (compare with Fig 2P-2Q). (U-V) Targeted overexpression of Rho produces dramatic effects on PPS morphology based on both abnormal Futsch-positive neuronal processes (V, asterisks) and the increase in Elav-positive nuclei. *z*-projected lateral views with anterior to the left and dorsal up.

Using *Rho654-Gal4* to drive expression of fluorescent reporter genes, we fate mapped the progeny of Dv1/3/4/7. During embryonic stages 11 and 12, the number of reporter-labeled cells increases (compare Fig 1C to Fig 2A). At the same time, these cells begin to migrate posteriorly along the ventral surface of the pharynx and a subset continues to express DPax2 (Fig 2A–2C). Consistent with the cells initiating *rho* expression and EGF secretion, activated MAPK (pERK), an effector of EGF signaling [14], is observed in and around these cells (Fig 2B and 2C). By the conclusion of embryogenesis, Rho654-labeled cells form two bilaterally symmetric domains. The smaller domain lies within the most anterior portion of the CNS and contains cells expressing the pan-neuronal marker Embryonic lethal abnormal vision (Elav, [34,35]), while the larger domain lies outside of the CNS and contains a subset of cells that weakly expresses Elav (Fig 2D).

The embryonic *Drosophila* brain is composed of three neuromeres (protocerebrum, deutocerebrum and tritocerebrum) that resemble the tripartite structure of the vertebrate brain [17]. Previous work using gene expression analyses correlated three-dimensional reconstructions of neuronal lineages in the late embryonic CNS with their neuroblast origins [22,36,37]. Comparing those reconstructions with the pattern of Rho654 reporter activity (Fig 2D and 2E), the labeled CNS domain appears to correspond to one or more of the dorsal anterior medial (DAM) lineages of the deutocerebrum.

The location of the larger, non-CNS domain (Fig 2D and 2E) suggests that those Elav-positive cells comprise the hypopharyngeal sensory organ (HPSO), a structure reportedly derived from the Dv1/3 neural precursors [17]. Consistent with a sensory neuronal identity, subsets of YFP-positive cells within this domain express a reporter of past *ato* activity [38] (Fig 2F), DPax2 (Fig 2G) and Eyes shut (Eys, [39,40]) (Fig 2H). Furthermore, expression of Eys is lost in the HPSO of embryos lacking *rho* (*rho* ^*7M43*^, [41], Fig 2I and 2J) or *spitz* (*spi* ^*1*^, [42], Fig 2K and 2L), the EGF ligand cleaved by Rho, suggesting that Rho plays a role in specification of cellular subsets within the HPSO. Other enhancer-labeled, Elav-negative cells surrounding the HPSO likely correspond to epithelial cells of the pharyngeal wall in which the HPSO is embedded. A similar pattern of HPSO and CNS expression was observed with a reporter of RhoB activity and subsets of HPSO cells were co-labeled by various sensory organ markers (S1 Fig). No expression was observed in the HPSO or CNS using a RhoA reporter (data not shown). Thus, the locations and expression profiles of these populations are consistent with the larger domain corresponding to the HPSO and containing progeny of the Dv1/3 SOPs [17,32,43] and the smaller domain comprising a neuronal lineage within the deutocerebral CNS composed of Dv4/7 neuroblast progeny.

We extended our fate mapping of Rho654 cells by examining reporter activity at later developmental stages. Reporter expression is not detectable in first instar larvae but reappears in second and third instar larvae in a region of the pharyngeal epithelium near the cuticular ridges. This region lies adjacent to the posterior pharyngeal sensilla (PPS, [44]), the larval counterpart to the embryonic HPSO (Fig 2M–2Q). Reporter activity is also seen in a small population of cells located near the midline in the supraesophageal region of the CNS and a network of projections in the ventral nerve cord (Fig 2R). Analysis of RhoA and RhoB reporters in larvae indicates that only RhoB promotes activity in the pharyngeal regions and neither of these reporters drives expression in the CNS (data not shown). These data suggest that the Rho654 reporter activity observed in third instar larvae results from reactivation of the RhoB region of the enhancer in the pharyngeal region and that the activity observed in the CNS is distinct and unrelated to that seen in the embryonic CNS. In addition, we observed reporter activity in a variety of sensory regions in the adult (data not shown). These include the internal pharyngeal sensory organs, eye, antenna, maxillary palps and labellum of the head as well as cells of the legs and wings. Due to the complexity of this activity pattern, a full characterization of Rho654 activity in the adult is ongoing and will be described in future work.

Having observed that Rho-dependent EGF signaling plays a role in the development of the HPSO, we next asked whether it also influences development and or maintenance of the PPS. Since both *rho* ^*7M43*^ and *spi* ^*1*^ are lethal, we instead used *Rho654-Gal4* to overexpress *argos (aos)*, an EGF receptor antagonist that impedes signaling by sequestering Spi [45]. Enhancer-targeted Aos overexpression produced only a modest decrease in GFP reporter activity but a significant change in PPS morphology. Analysis of the neural marker Futsch [46,47] in third instar *Rho654>Aos* larvae reveals that the PPS neuronal processes, which normally appear tightly bundled around the Elav-positive nuclei, instead appear “unwound” and terminate more distantly (Fig 2S and 2T). Strikingly, targeted overexpression of Rho results in even more profound defects in PPS morphology as evidenced by both abnormal Futsch-positive neuronal patterning and an approximate doubling of the number of Elav-positive nuclei (Fig 2U and 2V). Taken together, these results suggest that Rho-dependent EGF signaling plays a role in the development and/or maintenance of the PPS and that the regulation of signaling levels is key for proper morphology.

### Targeting toxin expression to neural precursors in the embryonic head impairs larval development and adult viability

**Fig 3:**
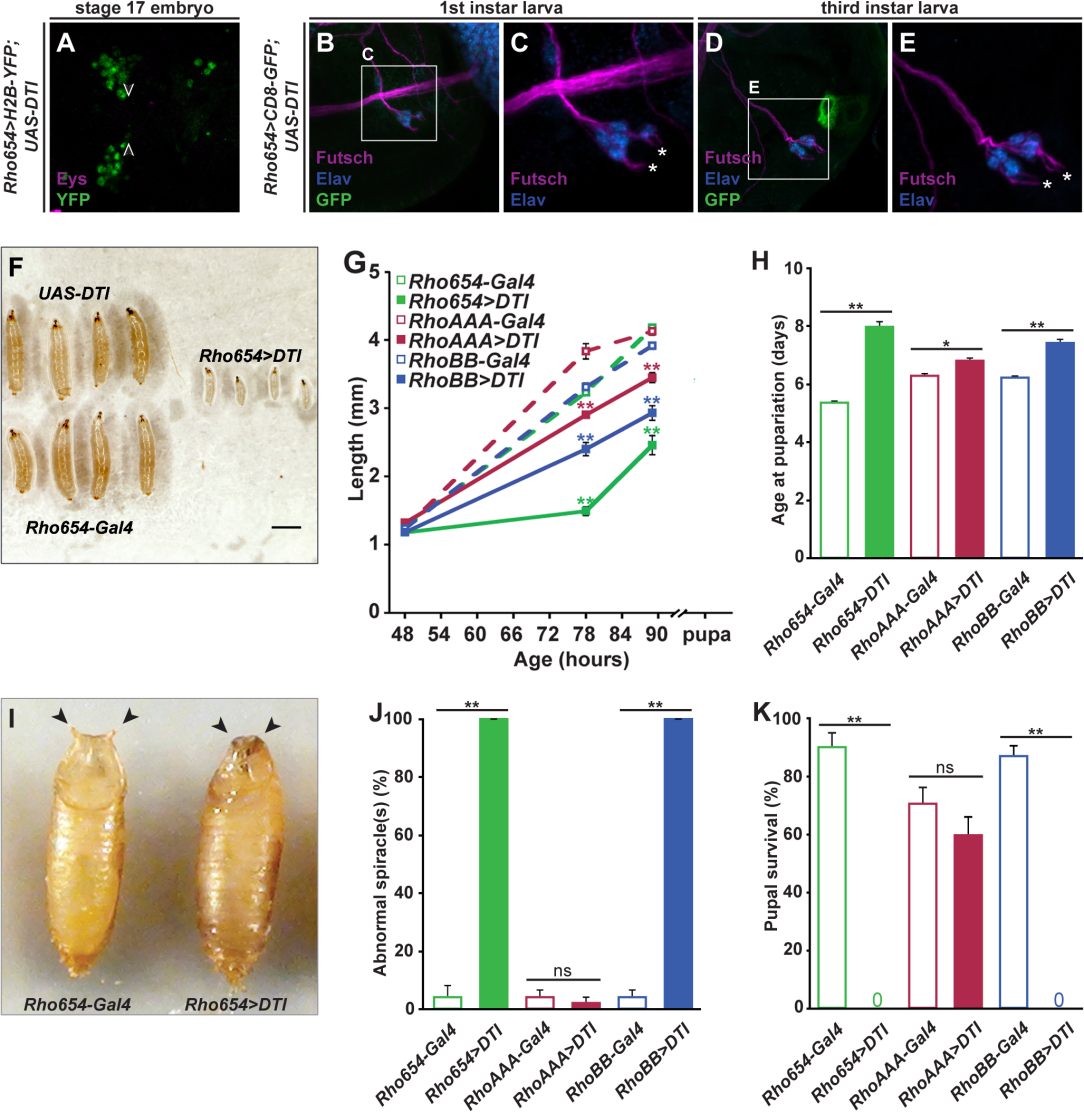
Enhancer-targeted toxin expression impairs development and viability. (A) Eys expression is not detected in the HPSO of toxin-targeted embryos despite the persistence of reporter activity in Rho654-defined cells. Open arrowheads denote normal location of Eys-expressing cells (compare to Fig 2H). Stage 17, *z*-projected dorsal view. (B-E) Reporter expression is undetectable (B) or reduced (D) in the PPS/pharyngeal region of toxin-targeted larvae and the relative position of Futsch-positive PPS neuronal termini (asterisks, C and E) is altered (compare Fig 2M–2Q). *z*-projected lateral views. (F) Comparison of control and toxin-targeted larvae at 72 hours AEL. Scale bar, 1 mm. (G) Quantification of length over larval/pupal time. (H) Age at pupariation for control and targeted flies. (I) Comparison of anterior spiracles in control and targeted pupae. (J) Quantification of abnormal anterior spiracle eversion. “Abnormal” denotes incomplete or absent eversion of at least one spiracle. (K) Pupal survival to adulthood. *p<0.01 or **p<0.001.

Despite the vital developmental role of neural precursors, characterization of the functional significance of individual precursors has been largely hampered by a lack of tools to link specific cells to their later roles. To examine the functional role of Rho654-defined cells, we used the Gal4/UAS system [48] to selectively express the diphtheria toxin A chain (DTI) [49,50]. This catalytic fragment inactivates the translation factor EF-2 causing arrest of protein synthesis [51]. To assess the efficacy of this toxin targeting scheme, we examined Rho654 reporter activity and marker gene expression over the course of development. Consistent with DTI expression requiring hours to days to achieve full efficacy [52,53], reporter expression appears largely unchanged in the HPSO of late stage embryos subject to toxin expression (Fig 3A). Similar to *rho* and *spi* mutants, however, expression of Eys is lost suggesting that Rho-dependent cell recruitment/specification has been disrupted. Reporter expression is largely undetectable in the PPS/pharyngeal region of toxin-targeted first instar larvae as it is in controls (Fig 3B and 3C). In contrast, however, reporter expression remains low or undetectable in third instar targeted larvae suggesting that toxin expression either ablates the Rho654-defined cells or severely compromises their function (Fig 3D and 3E). Furthermore, analysis of Futsch-labeled neuronal processes reveals defects in PPS morphology at all larval stages. Thus, enhancer-targeted toxin expression results in loss of Eys-positive cells in the embryonic HPSO as well as morphological changes in the larval PPS.

Further examination of *Rho654>DTI* flies reveals defects at multiple stages of development. First, a drastic reduction in size is observed by 72 hours after egg laying (Fig 3F). Quantification of length as a function of time reveals that toxin-targeted larvae show little increase in size between 48 and 72 hours in marked contrast to the rapid growth of control larvae (Fig 3G). After 72 hours, the growth rate of toxin-targeted larvae increases. While toxin-targeted larvae are capable of pupariation, they form significantly smaller pupae than controls (Fig 3G) and do so on average more than 2.5 days later (Fig 3H). We then asked whether these effects could be linked to the loss of Rho654-defined cells in particular body regions by selectively targeting cells in either the abdomen (*RhoAAA>DTI*) or head (*RhoBB>DTI*). While toxin expression in either region alters growth and increases the duration of development, the magnitude of these effects is greater with head cell expression and more similar to the deficits seen with Rho654 (Fig 3G and 3H). Examination of pupae revealed that, in addition to being smaller than controls, nearly all of those subject to head cell-targeted toxin expression (mediated by either *Rho654>DTI* or *RhoBB>DTI*) also exhibit defects in anterior spiracle eversion (Fig 3I and 3J). Such defects are rarely seen in control pupae or in pupae subject only to targeting of abdominal cells (*RhoAAA>DTI*). Finally, we assayed survival of toxin-targeted pupae and found that abdomen-specific targeting has little effect on viability but targeting of head cells results in pupal lethality (Fig 3K). The lack of lethality seen with abdomen-specific toxin expression is somewhat surprising since RhoA acts in SOPs that promote specification of oenocytes, a cell type essential for viability [13,16,18,54]. Nevertheless, we find no difference in oenocyte numbers in toxin-targeted embryos compared to controls and no gross changes in oenocyte appearance or location (S2 Fig), suggesting that the lag time inherent in the Gal4/UAS toxin expression protocol allows abdominal SOPs to secrete EGF and induce oenocyte specification prior to the onset of DTI expression. Taken together, these experiments demonstrate that the targeting of toxin expression to Rho654-defined neural precursors in the embryonic head results in severe defects in larval growth and pupation and ultimately leads to death prior to eclosion.

**Fig 4:**
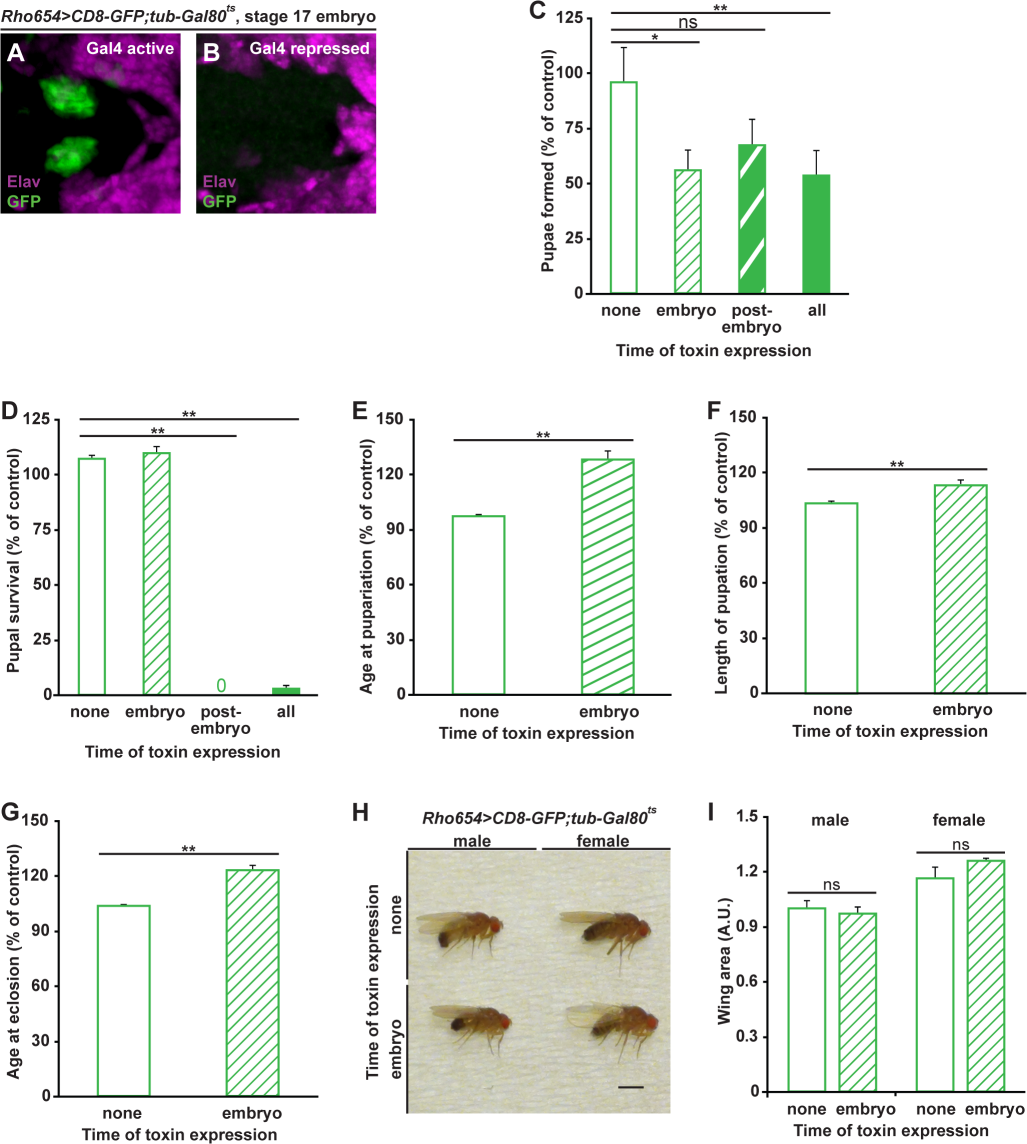
Temporal control of toxin expression separates defects in development and viability. (A-B) Temperature-sensitive Gal80 expression controls Gal4-dependent GFP reporter activity. Stage 17 embryos, *z*-projected dorsal views. (C-D) Quantification of pupae formed (C) and surviving to adulthood (D) for larvae subject to different toxin expression conditions. (E-G) Analysis of age at pupariation (E), length of pupation (F) and age at eclosion (G) for non-toxin-targeted and embryonically-targeted flies. Since developmental timing is temperature dependent, data are shown as experimentals (*UAS-DTI* positive) normalized to controls (*UAS-DTI* negative). *p<0.01 or **p<0.001. (H) Comparison of non-targeted and embryonically-targeted adults. Scale bar, 1 mm. (I) Quantification of wing area for non-targeted and embryonically-targeted flies. *p*>0.1 for inter-sex comparisons.

Since Rho654 acts during multiple stages of development and enhancer-mediated toxin expression results in phenotypes at more than one of these stages, we investigated whether there are critical periods for toxin expression associated with each phenotype. To introduce temporal control into the targeted toxin expression scheme, we used a temperature-sensitive version of Gal80 driven by a tubulin promoter (*tub-Gal80* ^*ts*^, [55]). At the permissive temperature (18°C), Gal80^ts^ represses Gal4 and prevents Gal4/UAS-mediated gene expression. At the restrictive temperature (29°C), Gal80^ts^ is inactive and Gal4/UAS-mediated expression occurs normally. After verifying that Gal4 activity is properly regulated at both the permissive and restrictive temperatures (Fig 4A and 4B), we compared the effects of toxin expression over four time windows: all of development, none of development, only embryogenesis and only post-embryogenesis. Embryonic expression results in nearly 50% fewer individuals reaching pupariation relative to those subject to no toxin expression, a number similar to that seen with expression over the entire course of development (Fig 4C). While post-embryonic toxin expression also leads to a decrease in the percentage of individuals reaching pupariation, the magnitude of this change is not statistically significant when compared to controls (Fig 4C, p>0.5). Of the larvae that do pupariate, nearly all of those subject to targeted expression solely during embryogenesis survive to adulthood while none of those targeted post-embryogenesis survive (Fig 4D). These findings suggest that enhancer-targeted toxin expression may result in death either before or after pupariation but different mechanisms are responsible for the two types of lethality. Since we observe enhancer activity in multiple regions of adults, we chose to focus on the effects of embryonic toxin targeting.

We next quantified age at pupariation and found that it increases by more than 20% with embryonic toxin expression (Fig 4E). Such expression also significantly increases the length of pupation (Fig 4F) and age at eclosion (Fig 4G). Intriguingly, however, adult survivors of embryonic targeting are of normal overall size (Fig 4H) and exhibit no significant difference in wing area compared to non-toxin targeted controls (Fig 4I). Thus, the observed phenotypes can be separated into two categories based on time of toxin expression; embryonic expression alters growth, delays development, and causes significant pre-pupal lethality while post-embryonic expression correlates with lethality at the pupal stage.

### Toxin expression in neural precursors alters feeding behavior and promotes abnormal satiation

Mutant [56,57] and targeted ablation [58] analyses have correlated developmental delay with an increase in adult size, an effect potentially explained by the increase in feeding time and expanded potential for growth associated with a lengthening of larval life. Thus, our observation that survivors of targeted embryonic toxin expression develop into adults of normal size despite an increase in the length of larval development, coupled with the finding that these larvae exhibit defects in growth, suggests that feeding might be affected. While there is no previous functional data for the PPS, its location and expression of taste receptors support the idea that it may play a role in feeding [59,60]. Feeding involves a variety of factors, including: (1) the ability to locate and move toward food, (2) the motivation to consume food, (3) the physical ability to ingest food and (4) the ability to metabolize and store food. We examined each of these factors in an effort to identify the cause(s) of the observed growth defect.

**Fig 5:**
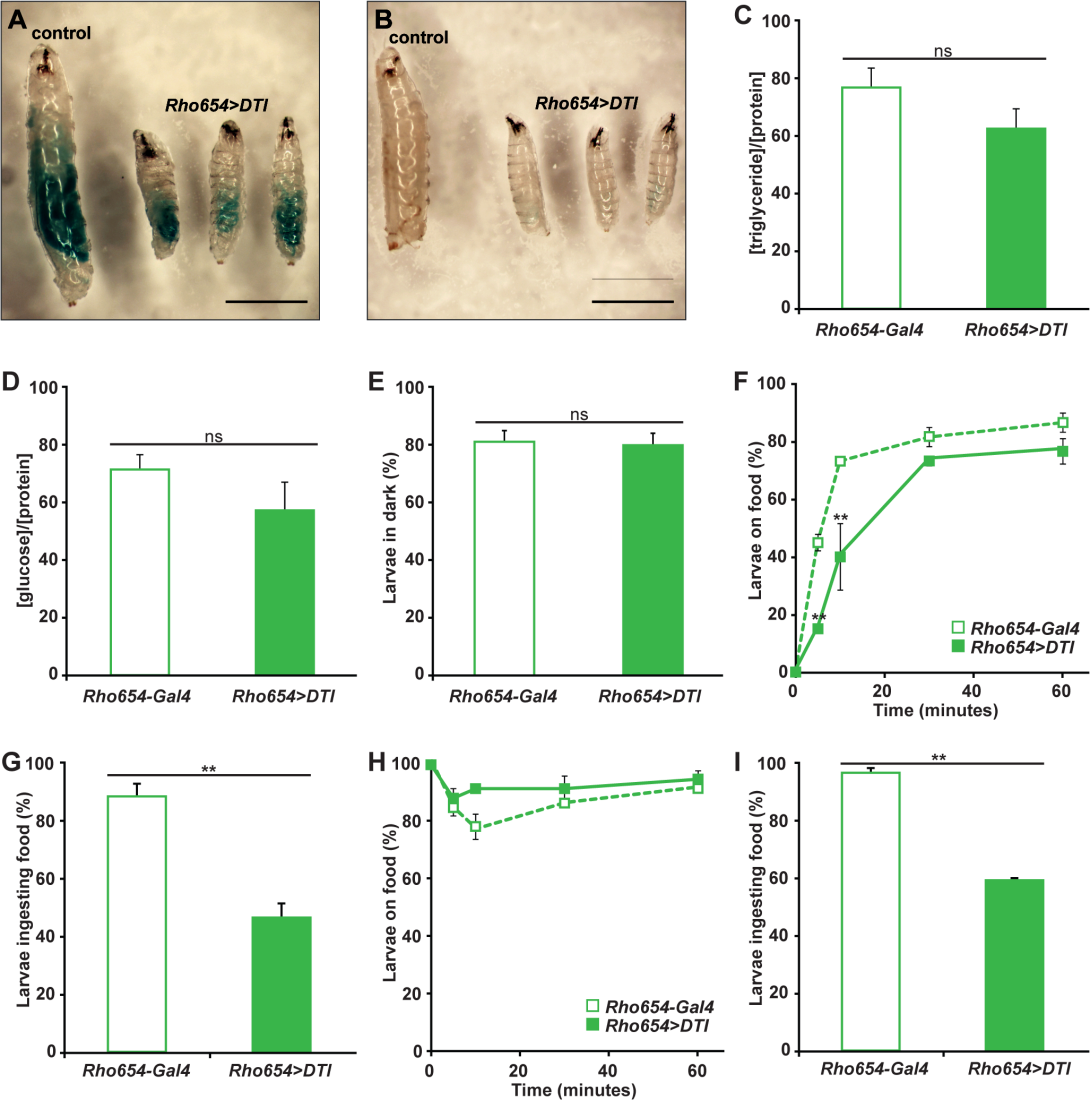
Enhancer-targeted toxin expression alters food consumption but not other measures of feeding competence. (A-B) Dyed food is visible in targeted larvae (A) indicating the capacity to ingest food and pump it through the gut. Two hours later, dye is no longer visible (B) demonstrating that the ability to clear food from the gut. (C-D) Concentrations of triglycerides (C) or glucose (D) normalized to protein concentration. *p*>0.1. (E) Percentages of *Rho654-Gal4* and *Rho654>DTI* larvae that display photophobicity. *p*>0.5. (F-I) Quantification of larval localization to a food source (F,H) and food ingestion after one hour (G,I). Larvae were initially positioned either distant from (F-G) or at the boundary of the food source (H-I). **p<0.001.

We first investigated whether the defect is correlated with changes in the capacity for food intake or metabolism. Examination of dyed food uptake revealed that targeted larvae are capable of food intake (Fig 5A) and clearance from the gut (Fig 5B). In addition, quantification of triglyceride (Fig 5C) and glucose (Fig 5D) levels shows no significant change in relation to total protein in targeted larvae compared to controls. Therefore, the growth defects observed with toxin expression are not explained by a physical abnormality that prevents food ingestion or clearance nor by changes in two commonly used measures of metabolic activity.

During analysis of food intake, we noted that toxin-expressing larvae displayed heterogeneous ingestion levels, leading us to ask whether alterations in feeding patterns could explain the growth and developmental defects. One external factor correlated with changes in feeding pattern is light preference. Throughout the first, second and early third instars, *Drosophila* larvae exhibit photophobicity and remain buried in their food source throughout the critical feeding period [61–63]. Later in the third instar, photophobicity is replaced by positive phototaxis as larvae cease feeding and begin wandering away from food prior to pupariation. As such, changes in larval phototaxis have been correlated with altered growth [64]. Toxin-expressing larvae display a normal pattern of phototaxis (Fig 5E), however, indicating that the growth phenotype is not the result of an improper response to light-based cues.

We next examined whether targeted toxin expression impairs the ability of larvae to detect and/or move toward a food source. To test for impairments in food detection, we located starved larvae at a distance from a food source and scored their movement toward the food over time. Fewer targeted larvae than controls reached the food at early time points, but after 30 minutes similar percentages were observed (Fig 5F). No abnormalities in the pattern of contractile movements were detected in targeted larvae, so we reasoned that their delayed arrival is a consequence of their smaller size and correspondingly slower speed (a conclusion borne out by later experiments). After a one-hour feeding period, we qualitatively examined food uptake and observed that 88% of controls displayed dyed food consumption compared to only 46% of toxin-targeted larvae (Fig 5G). Additionally, in most cases the quantity of food ingested by targeted larvae was noticeably less than that of controls even considering their difference in overall size. These findings suggest that the ability to detect and move toward food in a directed fashion is not impaired by targeted toxin expression but actual food consumption is decreased. To exclude the possibility that differences in food ingestion were a by-product of the increased time required for smaller targeted larvae to reach the food, we repeated the food detection assay but placed the larvae directly at the boundary of the food source. While percentages of control and targeted larvae located on the food were similarly high and relatively constant over the course of the assay (Fig 5H), once again significantly fewer targeted larvae showed evidence of food consumption after one hour (Fig 5I). Taken together, these results suggest that larvae subject to targeted toxin expression search for food normally but exhibit a decreased likelihood and/or magnitude of consumption.

**Fig 6:**
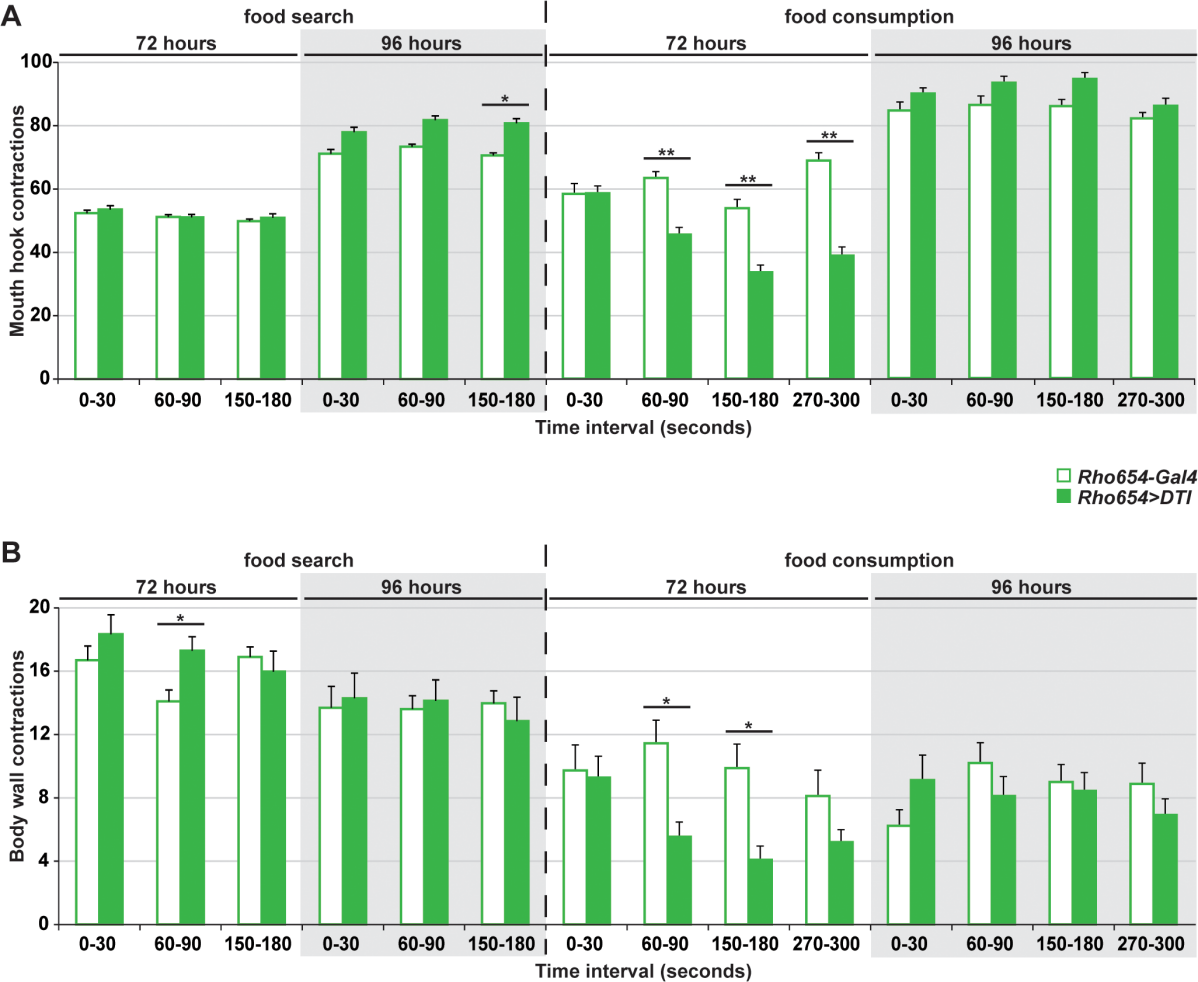
Enhancer-targeted toxin expression results in a temporary decrease in feeding behavior. (A-B) Quantification of mouth hook (A) and bodywall (B) contractions at 72 or 96 hours AEL. Contractions were scored for the same 25 larvae at 0–30, 60–90, 150–180 and 270–300 seconds after placement on test plate either distant from a food source (“food search”) or on a uniform food layer (“food consumption”). *p<0.01,**p<0.001.

To further characterize differences between food search behavior and actual consumption, we assayed mouth hook and body wall contractions in starved larvae placed either at a distance from a food source (“food search”) or on a uniform food layer (“food consumption”). Contraction rates were quantified for the same larvae over multiple 30 second intervals beginning immediately after placement (Fig 6). At both 72 and 96 hours of age, contractions rates were similar for control and targeted larvae in the food search condition and showed little variation between intervals, again implying that targeted toxin expression does not alter larval food search behavior. In the food consumption assay, however, contraction rates for 72 hour old targeted larvae were initially similar to controls but decreased significantly at subsequent intervals compared either to controls or to their own initial rates.

The rapid decrease in feeding exhibited by 72 hour old toxin-expressing larvae, but not controls, suggests two possible interpretations. First, the decreased rate observed in ablated larvae could be an indirect effect of developmental delay. To compare the developmental stage of 72 hour old toxin-expressing larvae with controls, we analyzed mouth hook morphology. While all control larvae were third instar (L3 = 100%; N = 28), the vast majority of ablated larvae were only second instar (L1 = 7%, L2 = 72%, L3 = 21%; N = 29). In contrast, nearly all 96 hour old ablated larva were third instar (L2 = 9%, L3 = 91%; N = 32). These data are consistent with a developmental delay in toxin-expressing larvae. To determine if the feeding defect observed in 72 hour old ablated larvae could be explained by differences in developmental stage, we analyzed mouth hook contractions for second instar control larvae (49 hours old, L2 = 100%; N = 19), which are of a similar size as ablated 72 hour old larvae (see Fig 3G). Importantly, we observe no significant difference in feeding rates between controls at 49 and 72 hours of age (data not shown), suggesting that differences in larval stage (i.e. developmental delay) cannot account for the altered feeding rate seen in 72 hour old targeted larvae.

A second possible interpretation for the decreased feeding rate observed for 72 hour old targeted larvae is that it is a direct effect of the toxin expression. Given that expression occurs in the area of a sensory organ already linked to feeding [59,60] and changes in the morphology of that organ are also observed in targeted larvae, we postulate that the decrease in feeding seen within one minute of food exposure results from alterations in the function of this organ leading to a satiety-like phenotype despite prior starvation. Overall, these data suggest that the altered pattern of growth and developmental delay seen with embryonic Rho654-targeted toxin expression is linked to an abnormal diminution of larval feeding behavior. This effect does not reflect a decrease in the motivation to find food but instead represents a failure to sustain intake once food is found. Strikingly, however, this effect is both temporary and reversible, implying that between 72 and 96 hours of age a change occurs which promotes the resumption of normal feeding.

## Discussion

The proper regulation of animal growth is dependent upon the complex interplay of metabolic, hormonal and neuronal pathways. While populations of neurons have been identified in the mature brain that are required for regulating growth in *Drosophila*, the lack of specific genetic tools to match many of these neurons with their precursors of origin has hindered studies linking development to function. Moreover, the lack of any functional data for other neuronal populations leaves open the possibility of finding additional regions that are critical for growth regulation. Our data addresses each of these issues by tracking a novel set of neural precursors through development and demonstrating that disrupting these precursors via targeted toxin expression produces measurable deficits in feeding and growth.

Building on our previous finding that the Rho654 *rhomboid* enhancer acts in distinct regions of the embryo [16], we used a fluorescent reporter to lineage-trace a population of cells from their origins through migration and subsequent differentiation. Initially, these cells comprise a set of four neural precursors located in the anterior region of the stage 11 embryo and, consistent with previous gene expression and mapping data for these precursors [17,20,23–25], give rise to two structures in the late embryo: a subset of cells in the deutocerebral portion of the CNS and a domain of pharyngeal cells which includes the HPSO, a sensory organ of the PNS. Importantly, we show that targeting toxin expression to these cells results in impairment of larval growth, delayed pupariation and pupal lethality. In addition, toxin-expressing larvae exhibit an age-dependent and reversible alteration in feeding behavior. Taken together, these results are significant for two reasons. First, they represent an experimental paradigm to study the function of precursor cells that give rise to previously under-characterized neuronal populations. Second, through an array of behavioral assays, we demonstrate that defective growth regulation correlates with a rapid abnormal diminution of larval feeding and locomotion, behaviors reminiscent of satiety.

### Enhancer activity facilitates fate mapping of neurons from embryo to adult

The *Drosophila* central and peripheral nervous systems arise from a large number of neuroblasts and SOPs in the early embryo. Lineage tracing studies have contributed to a broad understanding of which neuroblasts give rise to neuronal populations in the *Drosophila* brain [22,36,37,65–67] but the functions of many of these lineages remain largely unknown. Here, we exploit the activity of the Rho654 enhancer, and in particular that of its RhoB region, to follow the progeny of four neural precursors and demonstrate that they give rise to the HPSO and surrounding pharyngeal epithelial cells as well as a small subset of cells in the CNS. Two of these neural precursors express the proneural gene *ato*. Data from other regions of the embryo, most notably the abdominal SOPs, indicate that *ato* expression leads to Rho-dependent EGF signaling and cell recruitment [68,69]. Our results are consistent with this since mutation of *rho* or *spi*, the EGF ligand it cleaves, results in loss of Eys-positive cells in the HPSO. The HPSO is thought to correspond to the larval PPS [44] and we demonstrate that Rho654-targeted overexpression of *rho* leads to specification of excess Elav-positive PPS neurons. Furthermore, the PPS is maintained through metamorphosis to become the adult dorsal cibarial sense organ [70–72] and our preliminary characterization of Rho654 reporter activity in the adult head indicates labeling of this organ (data not shown). Thus, our reporter constructs provide a valuable tool for following a restricted set of neural precursors from their embryonic origins throughout the entire course of development and examining the effects of altered EGF signaling on specific neural structures.

Previous studies suggested potential functions for the HPSO/PPS based on its location. Keilin initially identified two circular depressions with regular borders on the ventral posterior region of the mouth hooks that were conserved across many species of cyclorrhaphan larvae [73]. He further observed that the depressions extended through the mouth hook to the underlying pharyngeal region and, while he could not actually detect a nerve branch running through the depression, postulated that this structure was evidence of a specialized sensory organ. This organ, which Keilin and later Hertweck [74] referred to as “Organ X”, was subsequently characterized in the embryo through examination of neural and proneural gene expression patterns and termed the HPSO [43,75,76]. Expression of *asense* was reported in cells of the HPSO at stages 13 and 14 as well as Futsch immunoreactivity at stage 15 indicating connectivity to the labral nerve [43]. This connectivity is maintained in the larval successor to the HPSO, the PPS [44], whose location in the lateral wall of the pharynx agrees well with Keilin’s original characterization.

### Targeted toxin expression reveals a complex combination of phenotypes

The restricted activity pattern of Rho654 allows for genetic manipulations aimed at elucidating function and the results of our enhancer-targeted toxin expression scheme were both dramatic and complex. Targeted expression results in a high level of pre-pupal lethality, severely retarded larval growth and delayed pupariation. In addition, while approximately half of targeted individuals succeed in surviving to pupal stages, all pupae die prior to eclosion. Altering the timing of toxin expression revealed that different mechanisms are responsible for the lethality observed at these two developmental stages. Furthermore, the fact that embryonic expression resulted in a variable phenotype (lethality in some individuals but merely growth and developmental timing defects in others) indicates that there is significant plasticity in the degree of impairment.

Our current genetic tools do not allow us to restrict toxin expression solely to precursors of the HPSO versus those of the CNS cell subset. In the course of this study, we investigated a number of more complex targeting schemes (such as the split Gal4 driver system [77] or use of Gal80 to suppress Gal4-mediated expression in either the CNS or HPSO) but the early onset of Rho654 activity made these intractable. As such, it is not possible to definitively link either of these areas with the observed defects. Furthermore, the lack of distinctive markers other than enhancer-dependent reporter activity to label the entirety of these two regions makes it difficult to determine whether the loss of reporter activity between late embryonic and early larval stages results from cell death or normal enhancer down-regulation. The persistence of PPS neurons in ablated larvae implies that full ablation of the sense organ did not occur though the alteration in cellular morphology suggests that toxin expression did adversely affect organ development and could account for the variability in severity observed for some phenotypes.

Despite these technical limitations, we propose that the sense organ is the primary mediator of the observed phenotypes for several reasons. First, between embryonic and larval stages, the CNS is known to undergo significant cell death and remodeling whereas previous characterization of the sense organ indicates that it is maintained largely unchanged even into adulthood [44,70–72]. Limiting toxin expression to embryogenesis is sufficient to recapitulate phenotypes observed at later developmental stages, suggesting that the relevant structures may be retained from embryo to larva. Second, Rho654 activity is not observed in first instar larvae, and the reappearance of reporter labeling seen in the CNS of third instar larvae is not due to the RhoB region of the enhancer that mediates activity in the embryonic CNS cell subset. As such, the reporter expression seen in the third instar CNS is likely the result of de novo enhancer activity in reactivated neuroblasts, similar to the activity observed in additional previously unlabeled sensory areas of adults. As such, these cells would represent a different population from the CNS neurons labeled in the embryo and therefore would not be capable of mediating the observed feeding and growth phenotypes. Finally, as stated previously, we observed distinct morphological defects in the PPS as a result of toxin expression. While the PPS has not been definitively linked to feeding via functional data, the organ’s location and expression of taste receptors [59,71] make it a good candidate for sensation of food intake and/or quality. For all of these reasons, we believe that alteration of the sense organ, rather than the CNS, is the most likely explanation for the defects observed in feeding and growth.

### Targeted toxin expression alters the motivation to feed promoting abnormal satiety

In this study, we used a variety of larval behavioral assays to uncover feeding defects in Dv1/3/4/7 neural precursor-targeted toxin-expressing animals. Comparison with other flies that demonstrate feeding defects suggests that none phenocopy the defects observed in the Dv1/3/4/7 targeted larvae. For example, mutation of *pumpless* [78], a putative homolog of the vertebrate glycine cleavage system, produces a reduction in growth similar to that seen with Rho654-mediated toxin expression. That defect, however, was correlated with cessation of feeding due to an inability to pump food from the pharynx into the esophagus, a phenotype we do not observe with Dv1/3/4/7 targeting. Similarly, loss of Neuropeptide F (NPF), the *Drosophila* homolog of the mammalian Neuropeptide Y, results in decreased larval feeding [79]. However, feeding cessation in NPF-deficient larvae is correlated with hypermobility and active movement away from a food source, behaviors normally associated with older larvae in preparation for pupariation. In contrast, Dv1/3/4/7 toxin-targeted larvae remain in contact with the food source and exhibit reduced locomotion.

Importantly, Dv1/3/4/7 targeted larvae initially respond normally to a short period of starvation by actively searching for food, but upon finding it, their rate of food intake and movement diminishes extremely rapidly. This diminution is at odds with the pattern of voracious feeding and movement seen in age-matched control larvae, suggesting that targeted larvae lose their motivation to continue feeding and undergo abnormal satiation. While a variety of recent studies have examined satiety in adult *Drosophila* [5–10] using proboscis extension reflex (PER) and capillary feeder (CAFE) assays, these are not applicable to larvae. Nichols examined the effects of Drosulfakinin, a *Drosophila* homolog of the vertebrate satiety-related peptide hormone cholecystokinin [80], in both larvae and adults using a direct application protocol and found a decrease in gut contractions at both developmental stages. Significantly, these effects were observed within one minute of application, a time scale similar to what we observed for decreases in feeding behavior in toxin-targeted larvae following food exposure. Moreover, in larvae, but not adults, the Drosulfakinin-mediated effect was transient and contractions returned to near control levels within three minutes of application. This suggests not only that larvae may experience satiety-like effects but also that such effects are modulated on a faster time scale than in adults, perhaps reflecting the critical importance of feeding to the timely progression of larval development. The limitations of such a direct application protocol for measuring larval satiety, however, are two-fold. First, it aims to measure the effects of a specific molecule but the applied dosage may not accurately reflect the change in endogenous levels produced by actual feeding. Second, since the protocol requires that larvae be fixed in place for the duration of the experiment, it quantifies only an indirect measure of feeding (gut contractions rather than mouth hook contractions or amount of food consumed). Our characterization of satiety using mouth hook contraction rate represents an improvement on this direct application protocol because it characterizes feeding behaviors in fully behaving animals and reflects physiologically relevant effects. Furthermore, unlike feeding assays performed only at a single timepoint, quantification at multiple timepoints allowed us to document both rapid (seconds) and long-term (hours) changes in behavior. Thus, our results demonstrate what may be the first physiologically relevant evidence for satiety-like effects in larvae and indicate that these effects occur on a remarkably fast timescale.

## Supporting Information

S1 Fig Additional genetic markers of reporter-labeled HPSO and CNS cells. (A-F) *RhoBB>H2B-YFP* immunostaining showing labeled CNS neurons (dashed outlines) co-localize with Elav (A) and Prospero (Pros, B) [91]. Subsets of HPSO cells (solid outlines) express Elav (A, low levels), Pros (B), DPax2 (C), Eyes shut (Eys; D) [39,40], Futsch (E) [46,47] and a *LacZ* reporter of past Senseless (Sens) activity (F) [92]. Stage 17, *z*-projected dorsal views. Panels A and D show the same embryo as do panels C and E.
(TIF)

S2 Fig Enhancer-targeted toxin expression does not alter oenocyte number. (A-G) *Rho654-Gal4* (A), *Rho654>DTI* (B), *RhoAAA-Gal4* (C), *RhoAAA>DTI* (D), *RhoBB-Gal4* (E), *RhoBB>DTI* (F) and *UAS-DTI* (G) embryos immunostained for the oenocyte marker Hepatocyte nuclear factor 4 (Hnf4). Stage 17, *z*-projected lateral views. (H) Quantification of oenocyte numbers per abdominal segment and per half embryo. Ten embryos were scored for each genotype. *p*>0.05 for per embryo and per segment comparisons using one way ANOVA with planned comparison of means.
(TIF)
