# Supplementary figures and images for: Rhomboid Enhancer Activity Defines a Subset of Drosophila Neural Precursors Required for Proper Feeding, Growth and Viability
Source: PLoS One. 2015 Aug 7;10(8):e0134915. doi: 10.1371/journal.pone.0134915 (PMC4529294; doi:10.1371/journal.pone.0134915)

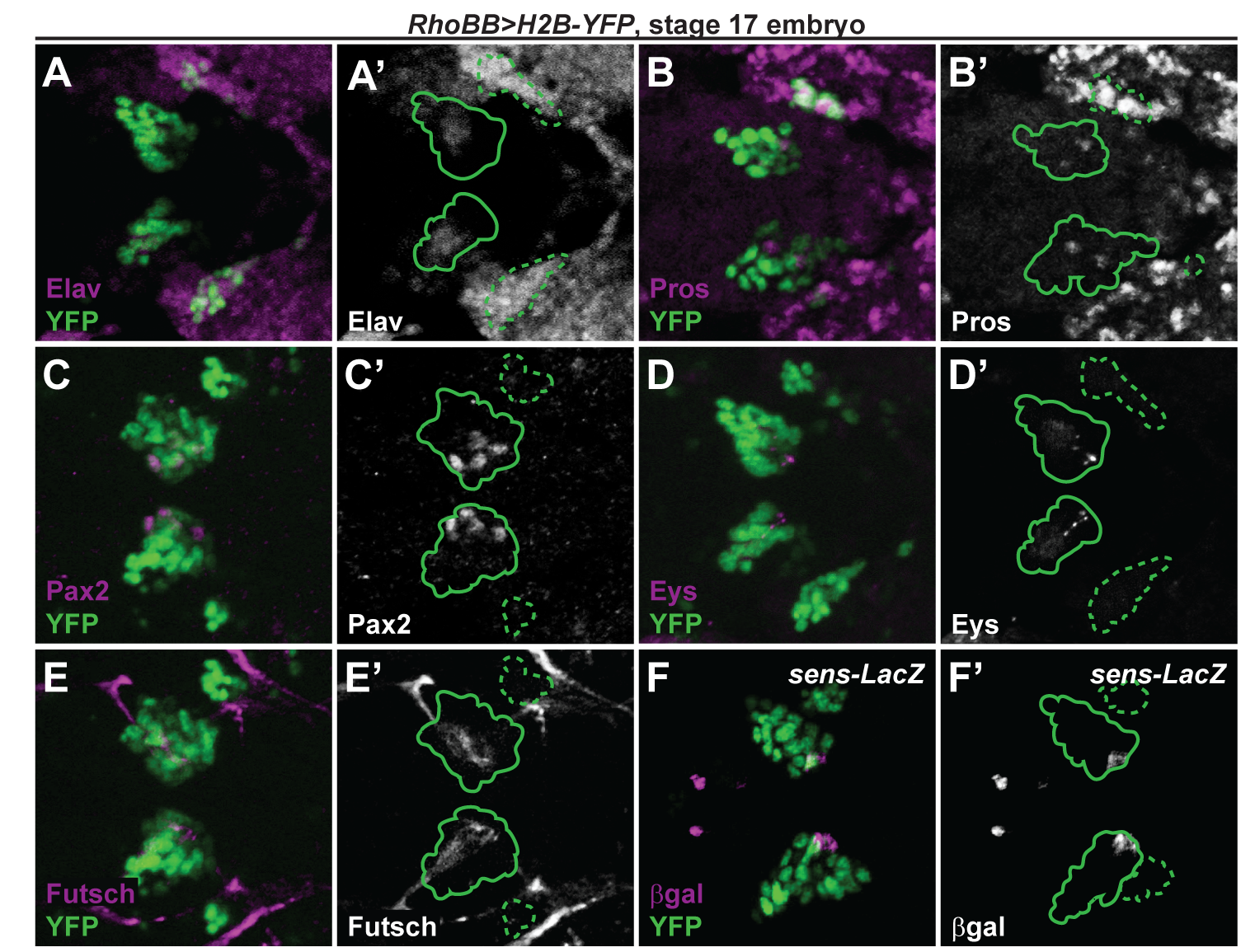

Supplement: S1 Fig — (A-F) RhoBB>H2B-YFP immunostaining showing labeled CNS neurons (dashed outlines) co-localize with Elav (A) and Prospero (Pros, B) [91]. Subsets of HPSO cells (solid outlines) express Elav (A, low levels), Pros (B), DPax2 (C), Eyes shut (Eys; D) [39,40], Futsch (E) [46,47] and a LacZ reporter of past Senseless (Sens) activity (F) [92]. Stage 17, z-projected dorsal views. Panels A and D show the same embryo as do panels C and E. (TIF) [file pone.0134915.s001.tif]

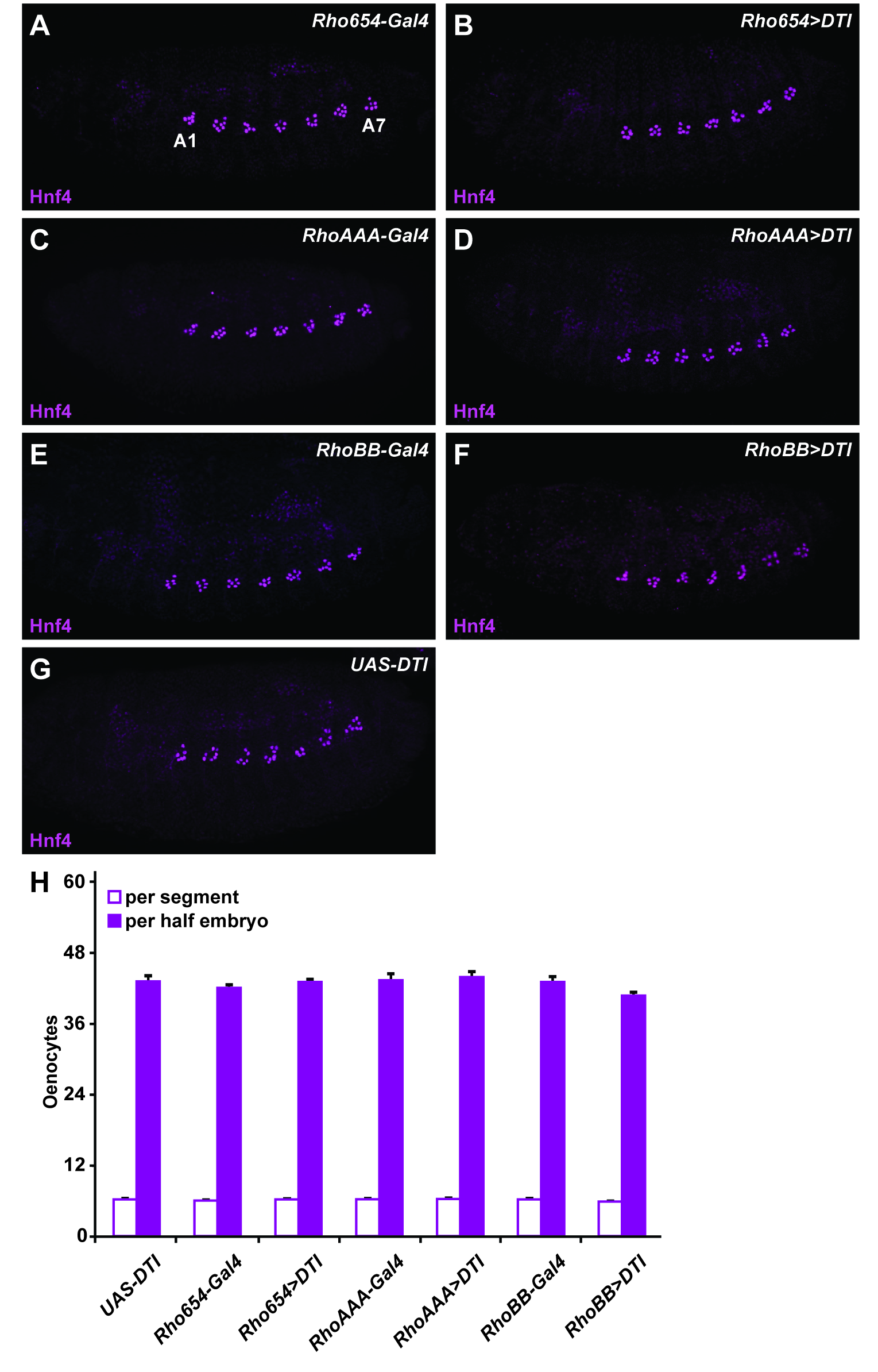

Supplement: S2 Fig — (A-G) Rho654-Gal4 (A), Rho654>DTI (B), RhoAAA-Gal4 (C), RhoAAA>DTI (D), RhoBB-Gal4 (E), RhoBB>DTI (F) and UAS-DTI (G) embryos immunostained for the oenocyte marker Hepatocyte nuclear factor 4 (Hnf4). Stage 17, z-projected lateral views. (H) Quantification of oenocyte numbers per abdominal segment and per half embryo. Ten embryos were scored for each genotype. p>0.05 for per embryo and per segment comparisons using one way ANOVA with planned comparison of means. (TIF) [file pone.0134915.s002.tif]
